# Predicting the potential demographic impact of predators on their prey: a comparative analysis of two carnivore–ungulate systems in Scandinavia

**DOI:** 10.1111/j.1365-2656.2011.01928.x

**Published:** 2012-03

**Authors:** Vincenzo Gervasi, Erlend B Nilsen, Håkan Sand, Manuela Panzacchi, Geir R Rauset, Hans C Pedersen, Jonas Kindberg, Petter Wabakken, Barbara Zimmermann, John Odden, Olof Liberg, Jon E Swenson, John D C Linnell

**Affiliations:** 1 Norwegian Institute for Nature ResearchPO Box 5685, Sluppen, NO-7485 Trondheim, Norway; 2 Department of Ecology and Nature Resource Management, Norwegian University of Life SciencesPO Box 10 5003, NO-1432 Ås, Norway; 3 Department of Ecology, Grimsö Wildlife Research Station, Swedish University of Agricultural ScienceSE-730 91 Riddarhyttan, Sweden; 4 Department of Wildlife, Fish, and Environmental Studies, Swedish University of Agricultural SciencesSE-901 83 Umeå, Sweden; 5 Faculty of Applied Ecology and Agricultural Sciences, Hedmark University CollegeEvenstad, NO-2480 Koppang, Norway

**Keywords:** *Alces alces*, *Canis lupus*, *Capreolus capreolus*, courser, kill rate, life history, *Lynx lynx*, stalker, *Ursus arctos*, *Vulpes vulpes*

## Abstract

**1.** Understanding the role of predation in shaping the dynamics of animal communities is a fundamental issue in ecological research. Nevertheless, the complex nature of predator–prey interactions often prevents researchers from modelling them explicitly.

**2.** By using periodic Leslie–Usher matrices and a simulation approach together with parameters obtained from long-term field projects, we reconstructed the underlying mechanisms of predator–prey demographic interactions and compared the dynamics of the roe deer–red fox–Eurasian lynx–human harvest system with those of the moose–brown bear–gray wolf–human harvest system in the boreal forest ecosystem of the southern Scandinavian Peninsula.

**3.** The functional relationship of both roe deer and moose λ to changes in predation rates from the four predators was remarkably different. Lynx had the strongest impact among the four predators, whereas predation rates by wolves, red foxes, or brown bears generated minor variations in prey population λ. Elasticity values of lynx, wolf, fox and bear predation rates were −0·157, −0·056, −0·031 and −0·006, respectively, but varied with both predator and prey densities.

**4.** Differences in predation impact were only partially related to differences in kill or predation rates, but were rather a result of different distribution of predation events among prey age classes. Therefore, the age composition of killed individuals emerged as the main underlying factor determining the overall per capita impact of predation.

**5.** Our results confirm the complex nature of predator–prey interactions in large terrestrial mammals, by showing that different carnivores preying on the same prey species can exert a dramatically different demographic impact, even in the same ecological context, as a direct consequence of their predation patterns. Similar applications of this analytical framework in other geographical and ecological contexts are needed, but a more general evaluation of the subject is also required, aimed to assess, on a broader systematic and ecological range, what specific traits of a carnivore are most related to its potential impact on prey species.

## Introduction

Understanding the contribution of predators in shaping the structure of ecological communities is a central issue in ecology. A plethora of diverse and often contrasting studies have been produced concerning the extent to which predators are able to limit the abundance of their prey species (Boutin 1992; Boertje, Valkenburg & McNay 1996; Atwood, Gese & Kunkel 2007).

Long-term studies of wolf–moose interactions in North America have long been the benchmark for large mammal predator–prey studies (Messier & Crête 1985; Messier 1994; Eberhardt 1997; Peterson 1999; Hayes & Harestad 2000), although data from several other systems have begun to accumulate (Gese & Grothe 1995; Molinari-Jobin *et al.* 2002; Laundré, Hernández & Clark 2006; Nilsen *et al.* 2009a). Despite such a strong research effort, there is still no general agreement on the degree to which predation influences prey population growth rate (λ), and especially on the mechanisms of such processes. Evidence exists for a weak influence of predation, especially when environmental productivity is high and predator numerical response is absent (Skogland 1991; Boutin 1992), whereas studies in other systems show that ungulate densities can be effectively limited by predation, especially if predators themselves are not controlled through harvest (Messier 1994; Eberhardt 1997) and when environmental productivity is low (Melis *et al.* 2009, 2010). Moreover, generalizations cannot easily be drawn when dealing with predator–prey interactions, as the potential a predator has to limit a given prey population is influenced by a variety of ecological factors, such as spatiotemporal variation in the availability of alternative prey species (Hebblewhite *et al.* 2003; Cooley *et al.* 2008), presence of other predators (Atwood, Gese & Kunkel 2007), predator–prey body size relationships (Sinclair, Mduma & Brashares 2003; Owen-Smith & Mills 2008), habitat heterogeneity (Gorini *et al.* 2011) and climate (Wilmers, Post & Hastings 2007). Local and short-term effects can also be influenced by stochastic variation in the predator's hunting success, as a consequence of individual heterogeneity (Festa-Bianchet *et al.* 2006). Such effects does not always contribute to the long-term demographic effect of predation, which might be determined by other factors. Therefore, attention is increasingly dedicated to understanding why some predators in certain situations seem to exert a stronger control on their prey than others (Atwood, Gese & Kunkel 2007), and most of all to identify the main predictors of a predator's potential impact on its prey species (Wilmers, Post & Hastings 2007; Owen-Smith & Mills 2008).

**Fig. 1:**
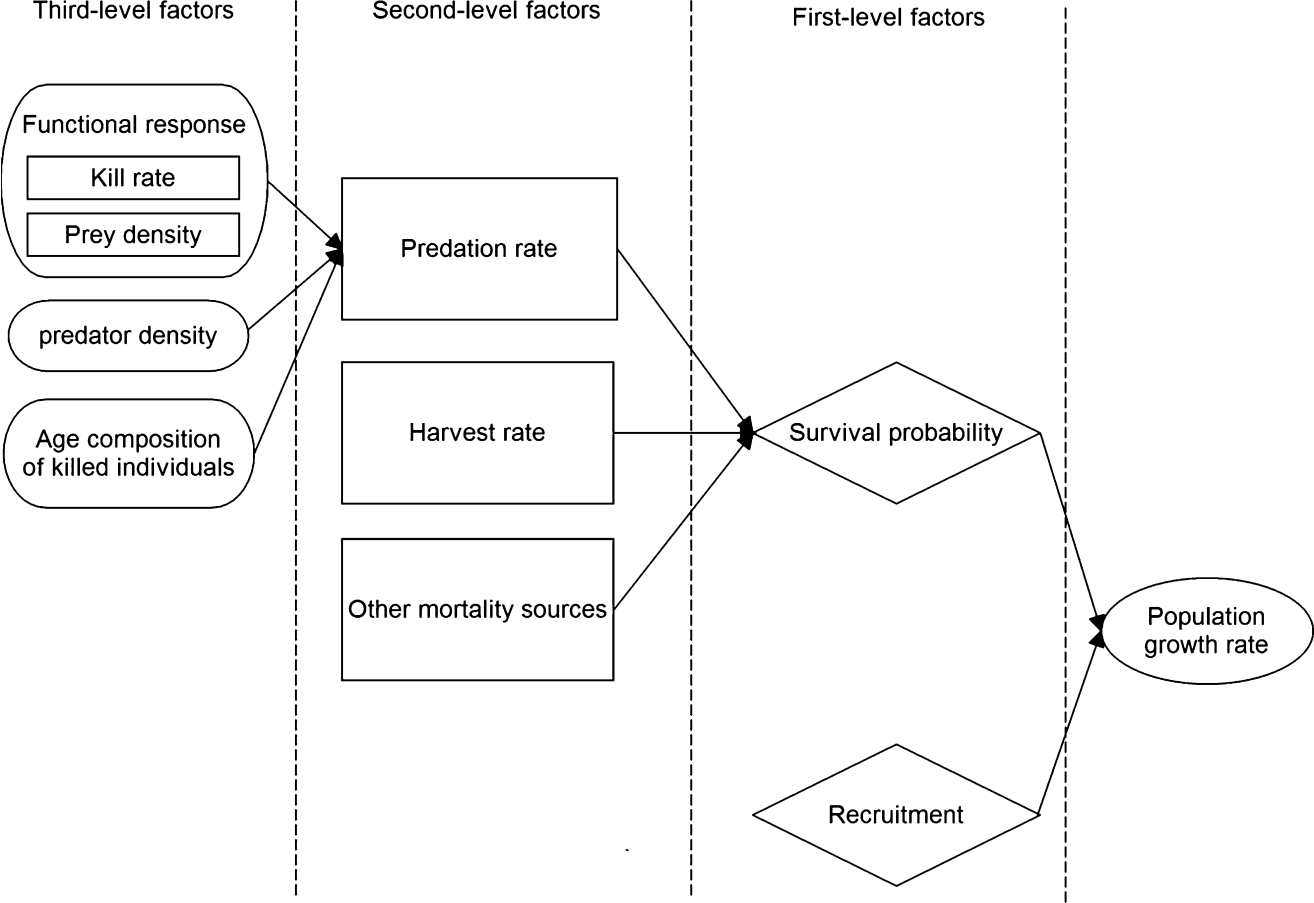
Structural diagram illustrating the demographic processes leading to the observed growth rate in prey populations, decomposed into three levels of increasing detail. Level 1 includes the effect of main demographic rates (age-specific survival, recruitment); in level 2, individual survival is decomposed into its basic mechanisms, that is, the main age-specific mortality factors (predation rate, harvest rate, natural mortality rate); in level 3, age-specific predation rate is decomposed into its basic mechanisms, that is, kill rate, predator density, prey density and age class of killed individuals.

The demographic process leading to the observed growth rate in prey populations can be decomposed into three nested levels of increasing complexity (Fig. 1):

1. At the simplest level, the overall annual growth rate of a population is determined by the contribution of both its reproductive and survival performances (Fig. 1, level 1), with adult survival usually retaining the highest functional relationship with population growth rate in slow-living species with long generation time, as large herbivores (Gaillard *et al.* 2000, 2005).
2. If the population is subject to several mortality factors, such as predation from different predators, human harvest or natural mortality, the overall mortality rate for each age class is the result of the combined effect of all these risk factors. Therefore, at a second, underlying level of complexity, the age-specific survival rates of the prey species can be decomposed into contributions from all the mortality factors acting on the population (Fig. 1, level 2). These factors can interact in an additive or partly compensatory way (Sandercock *et al.* 2011). In particular, the age-specific predation rate, that is, the proportion of prey population that is removed annually by a specific predator, reflects the predator–prey relationship at the population level.
3. At a third, more complex level, predation rates can also be decomposed into contributions from four basic factors: (i) per capita kill rate, which represents predation intensity, with functional response describing its relationship with prey density (Solomon 1949; Abrams & Ginzburg 2000); (ii) the age composition of killed individuals, which mainly reflects differences in the predation strategies of different predators, and in body size relationships between predator and prey (Sinclair, Mduma & Brashares 2003; Wilmers, Post & Hastings 2007; Owen-Smith & Mills 2008); (iii) predator density; and (iv) prey density. It is the interactive effect of these fundamental four factors which determines the overall age-specific predation rate, and thus the potential demographic impact of predation (Fig. 1, level 3).

In this theoretical framework, ecological theory suggests that non-selective stalking predators potentially exert a stronger demographic effect than more selective coursing predators, as they are able to remove a significant proportion of adults from the prey population (Sunquist & Sunquist 1989; Sinclair, Mduma & Brashares 2003). Moreover, at the population level, the extent of the demographic impact on ungulate populations is thought to be mainly affected by the predator/prey density ratio (Testa 2004) and by the predator's ability to respond numerically to changes in prey density (Messier 1994). Nevertheless, the number of parameters involved and the complexity of the interactions among them often prevent ecologists from completely reconstructing this demographic process, thus forcing them to indirectly assess the impact of predation by focusing only on one or two of the involved nested processes (Messier 1994; Hayes & Harestad 2000; Morrison & Hik 2007; Cooley *et al.* 2008; Nilsen *et al.* 2009a).

Here, we present the results of a comparative study on the potential demographic impact of two couples of mammalian carnivores, the Eurasian lynx *Lynx lynx* L., the red fox *Vulpes vulpes* L., the gray wolf *Canis lupus* L., and the brown bear *Ursus arctos* L., on two ungulate species, the roe deer *Capreolus capreolus* L. and the moose *Alces alces* L., in the boreal forest ecosystem of the southern Scandinavian Peninsula. We compared the structure and the dynamics of the *roe deer–red fox–Eurasian lynx* system with those of the *moose–brown bear–gray wolf* system. Such a study design allowed us to compare the impact of four very different predation patterns: during a short period in the summer, red fox and brown bear predation operates intensively on roe deer fawns and moose calves, respectively (Swenson *et al.* 2007; Panzacchi *et al.* 2008a, b), whereas during the rest of the year, fawns and calves are minimally affected by these two predators (Swenson *et al.* 2007; Panzacchi *et al.* 2008a, b). In contrast, all age classes in both populations are subject to harvest-related mortality risks during autumn. Lynx and wolves prey upon all age classes throughout the year, even though kill rates and the age composition of killed individuals differ between the two seasons (Sand *et al.* 2005, 2008; Nilsen *et al.* 2009b). These differences provided us with the opportunity to empirically compare the differential demographic impact inherent in each of the above-described predation strategies, namely that of a solitary stalking predator killing prey across a wide range of age classes (the Eurasian lynx), with that of a social coursing predator focusing on the most vulnerable juvenile class (the gray wolf), and with those of two facultative predators (the red fox and the brown bear) whose predation is mainly limited to the newborn segment of the prey population. Moreover, roe deer and moose are at the two extreme ends of the fast–slow continuum of ungulate life cycles (Gaillard *et al.* 2005), with the first exhibiting a much higher intrinsic growth rate and a lower sensitivity of population growth rate to changes in adult survival. This also allowed us to account for the variation in the relative importance of different vital rates along the above-cited continuum (Gaillard *et al.* 2000). In our case, the intensive demographic study of both predator and prey populations in Scandinavia over the last 25 years (Sand *et al.* 2005; Swenson *et al.* 2007; Panzacchi *et al.* 2008a, b; Cobben *et al.* 2009; Nilsen *et al.* 2009a, b; Linnell *et al.* 2010) allowed the estimation of an extensive set of predation and demographic parameters for these species, thus providing us with the unique opportunity to explicitly model their trophic and demographic relationships. Therefore, besides theoretically exploring the complexity and the multi-level nature of the demographic effects of predation, our study provides a powerful empirical test of the expectations regarding the role of predation strategy in determining the potential impact of a predator on its prey population. It should be noted that we did not try to estimate the actual extent of top-down control by each predator in our system, but rather to assess the potential demographic impact associated with each predator predation pattern.

We used a matrix modelling approach to reconstruct the complete underlying process of predator–prey demographic interactions, thus ultimately linking the specific predation patterns of each carnivore to the downstream population growth rate of its prey species. Moreover, as both carnivore and ungulate populations in Scandinavia are numerically limited through a system of adaptive harvest quotas, we also included human harvest as an additional demographic factor in the predator–prey system, thus assessing the relative impact of predation and harvest on the demography of roe deer and moose populations in a real-world management situation and in a multi-use, human-dominated ecosystem.

Therefore, based on the theoretical background exposed above and on the characteristics of the two predator–prey systems, we tested the following predictions:

1. The Eurasian lynx, being a non-selective stalking predator, exhibits a stronger per capita impact of predation on its prey than the gray wolf, the red fox or the brown bear.
2. The relative impact of the four predators at the population level significantly changes along a gradient of prey and predator densities.
3. The age composition of killed individuals is a better predictor of the per capita impact of predation than kill rate.

## Materials and methods

### Study Area

The studies from which field data were extracted were conducted in the trans-boundary boreal forest ecosystem of south-central Scandinavia, mainly within the Norwegian counties of Hedmark, Akershus and Østfold and the Swedish counties of Dalarna, Värmland, Västra Götaland and Örebro. Data on Eurasian lynx, roe deer and red fox were collected in Norway, data on bear predation on moose in Sweden and data on wolf predation on moose in both countries. The whole area is dominated by an extensive (but intensively managed) boreal forest consisting mainly of Norway spruce *Picea abies* L. and Scots pine *Pinus sylvestris* L., but there is a marked north–south gradient in the proportion of agricultural areas, which increases from *c.* 3% in the north to *c.* 21% in the southern parts of the study area. Elevations usually range from 200 to 300 m at the bottom of river valleys to 700–800 m on the Norwegian side; they are usually lower in Sweden. Roe deer and moose are present throughout the study area, with their densities following a north–south gradient, with lower densities in the northern areas (Lavsund, Nygrén & Solberg 2003; Panzacchi *et al.* 2008a, b). Mountain hares *Lepus timidus* L., black grouse *Tetrao tetrix* L., and capercaillie *Tetrao urugallus* L. are also present throughout the area. A few red deer *Cervus elaphus* L. and wild boar *Sus scrofa* L. are present in the western and southern parts of the study area, respectively, wild reindeer *Rangifer tarandus* L. are present on the very western edge of the study area in Norway, and free-ranging, unguarded domestic sheep *Ovis aries* L. are locally available as summer prey in minor parts of the Norwegian side of the study area. Prey switching, even though possible, was therefore a minor element of our system with respect to the wolves and the lynx, as they strongly rely on moose and roe deer for their diet (Olsson *et al.* 1997; Odden, Linnell & Andersen 2006). Foxes and bears are more generalist in nature, only routinely exploiting ungulate neonates in spring (Dahle *et al.* 1998; Panzacchi *et al.* 2008a, b). Carnivore numbers are regulated by harvest in both countries through a system of adaptive harvest quotas, so that no significant predator numerical response to fluctuations of prey densities is allowed. Baseline densities in the study area were of 0·57 and 1·00 individuals per km^2^ for roe deer and moose, respectively, and 0·4, 0·5, and 1·0 individuals per 100 km^2^ for lynx, wolf, and bear, respectively. More detailed study site descriptions, as well as details of field methods and density estimates, are presented in Swenson *et al.* (2007), Sand *et al.* (2008) and Nilsen *et al.* (2009a, b).

### Predation Patterns

For each of the four predators, we collected all the available information about per capita kill rate, functional response, predation rate and age composition of killed individuals to compile a comparative quantitative description of their predation patterns. Kill rates and a functional response equation were available for lynx (Nilsen *et al.* 2009b), wolf (Sand *et al.* 2005, 2008) and brown bear (Swenson *et al.* 2007), but not for red fox. Therefore, in the following analyses, we were able to compare the four predators in terms of all the main factors that defined their predation patterns, with the exception of red fox per capita kill rate.

### Roe Deer and Moose Life Cycles

Both roe deer and moose life cycles are characterized by two contrasting seasons, during which different mortality factors affect individual survival probabilities (see Introduction). Therefore, we structured our analysis around a two-season periodic life cycle (Skellam 1966) with three age classes (fawns: 0–12 months, yearlings: 1–2 years, adults: >2 years) for roe deer, and four for moose (calves: 0–12 months; yearlings: 1–2 years; 2-year-olds; adults: >2 years old; Gaillard *et al.* 2000). A pre-breeding life cycle was used for summer, assuming that population census occurred just before the breeding season each year, and thus included fawn/calf summer survival in the recruitment rate. The autumn–winter matrix contained survival probabilities only.

For each season and age class, individual survival probabilities were estimated as a function of the various mortality risks, according to the following expression (Skalski, Ryding & Millspaugh 2005):


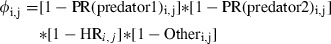
eqn 1

where

*ϕ*_i,,j_ = survival probability for an individual of age class i during season j

PR(predator)_i,,j_ = predation rate of a given predator on age class i during season j

HR_i,,j_ = harvest rate on age class i during season j

Other_i,,j_ = mortality probability from other risk factors affecting age class i during season j

In a subsequent decomposition of predator–prey relationships inside roe deer and moose life cycles, predation rate was estimated as a function of its fundamental predictors, as follows:


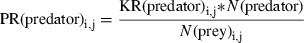
eqn 2

where

KR(predator)_i,j_ = per capita kill rate on age class i during season j

*N*(predator) = predator abundance

*N*(prey)_i,j_ = abundance of prey individuals of age i during season j

We were not able to produce the same function to calculate red fox predation rate, because both per capita kill rate and red fox abundance in the study area were not known. Therefore, red fox predation rate on roe deer fawns was modelled through a three-step discrete function, with increasing values of 0·10, 0·22 and 0·42, corresponding to three different levels of roe deer density (1·5, 3·0 and 15·0 roe deer per km^2^; J. Linnell unpublished data). Age-specific harvest rates for roe deer were also derived from J. Linnell (unpublished data), through the estimation of harvest-caused mortality probabilities in a sample of radio-collared roe deer of all age and sex classes (*N* = 299). Moose harvest rates were derived from Ericsson & Wallin (2001), who estimated cause-specific mortality rates from a sample of radio-collared moose in central-northern Sweden (*N* = 264). Other mortality probabilities, mainly related to natural and vehicular mortality risks, were fixed to 0·05 annually for both species (Ericsson & Wallin 2001, Cobben *et al.* 2009). For both species, we did not consider density dependence in vital rates, as both roe deer and moose densities in our study area were well below the levels at which density-dependent factors substantially affect survival and reproductive performances (Andersen & Linnell 2000; Nilsen *et al.* 2005, 2009a; Cobben *et al.* 2009). Also, the upper limit of prey densities used for simulations (see the Perturbation analysis section below) was well below prey-carrying capacity for both species. We also did not include any decreasing survival through senescence, as the combined effect of harvesting and predation in our study area reduces the proportion of senescent individuals to a negligible level for both species (Mysterud, Solberg & Yoccoz 2005; Nilsen *et al.* 2009a). Finally, we did not consider prey switching by predators as a model factor, as both roe deer and moose were by far the main prey species for wolves and lynx (Swenson *et al.* 2007; Sand *et al.* 2008; Nilsen *et al.* 2009b).

The main assumption in the roe deer and moose life cycle models was that predation and harvest mortality rates were additive with respect to other risk factors. We considered this assumption to be reasonable for our case, as several independent studies from the same ecological system found no evidence of relevant compensation between predation, human harvest and other causes of natural mortality in the two prey species (Nilsen & Solberg 2006; Andersen *et al.* 2007; Nilsen *et al.* 2009a). In Norway, roe deer survival was 35% lower in environments with human harvest and large predators (Nilsen *et al.* 2009a), compared to areas where both these factors were absent (Cobben *et al.* 2009), and Swenson *et al.* (1999) found that moose calves in areas with and without brown bears in Sweden had the same probability of dying because of other reasons than predation.

Following Nilsen *et al.* (2009b), who included the effect of lynx social status on per capita kill rates, lynx functional response was calculated separately for solitary individuals and family groups, thus accounting for the higher kill rates by females with kittens. For wolves, summer and winter kill rates were modelled separately, based on their differential predation patterns between the two seasons (Sand *et al.* 2005, 2008). In particular, winter functional response was derived from Sand *et al.* (unpublished data), whereas summer kill rates, as estimated by Sand *et al.* (2008), were applied assuming saturation of wolf predation in the range of simulated moose density (Sand *et al.* 2008). Brown bear kill rates were derived from Swenson *et al.* (2007), and no functional response was included in the model, as no change in brown bear kill rates on moose calves occurred within a range of moose densities from 0·5 to 1·5 moose per km^2^ (J. Swenson unpublished data). Based on the age/sex structure of the Scandinavian brown bear population (Swenson *et al.* 1994), only 50% of the bears were considered to be adults (>2 years old) actively predating on moose. Formulations and values for all the input parameters of both moose and roe deer life cycles are summarized in Table S1 and in Appendices S2–S3 (Supporting information).

### Matrix Analysis

Based on the structure of the life cycles described above, and on the corresponding demographic rates, a two-season periodic Leslie–Usher matrix (Caswell 2001) was constructed for the female segment of each of the two prey species, in which:


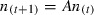
eqn 3

where *n*_*(t)*_ and *n*_*(t + 1)*_ are the population vectors in two successive years, and *A* is the projection matrix (Caswell 2001). In the particular case of a periodic projection matrix, in which the overall annual cycle is the result of two seasonal demographic processes, *A* is given by the matrix product of two seasonal matrices *B*_1_ and *B*_2_ (Caswell 2001). Therefore, the overall projection matrices for roe deer and moose populations, respectively, were given by:


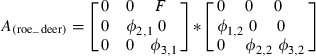
eqn 4

and


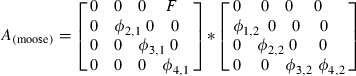
eqn 5

### Perturbation Analysis

The two Leslie–Usher matrices were used as a convenient starting point to perform a perturbation analysis of the main predation patterns, and consequently of demographic rates, in the two systems, thus assessing the functional dependence of λ on variation in these parameters. Predation impact was initially modelled at the baseline prey and predator densities reported for our study area (Sand *et al.* 2008; Nilsen *et al.* 2009b; Linnell *et al.* 2010). We used 0·54 and 1·0 individuals per km^2^ for roe deer (see Appendix S1 and Fig. S1) and moose, respectively, and 0·4, 0·5 and 1·0 individuals per 100 km^2^ for lynx, wolf and bear, respectively.

Then, perturbation analyses were performed around the baseline scenario at three levels, corresponding to the three levels of insight into the ecological relationships between predation patterns and prey demographic processes (see Fig. 1). At the first level, the overall elasticity of λ to variation in demographic rates (i.e. survival and recruitment) was calculated. Following Caswell (2001), elasticity is defined as the proportional change in λ, resulting from a proportional change in any of the vital rates included in a population matrix. In our case with periodic matrices, the overall elasticity values, derived from matrix *A*, do not provide a direct indication of the relative contribution of each demographic parameter to variation in λ. This is because each element of matrix *A* is the result of a complex mixture of the life-history traits in B_1_ and B_2_, which confounds the demographic interpretation of numeric values (Caswell & Trevisan 1994). Therefore, we applied the method described in Caswell & Trevisan (1994) to calculate the elasticity of λ to changes in the entries of each of the seasonal matrices of the model, using R 11.2.0 (R Development Core Team 2008) and the package popbio (Stubben & Milligan 2007) to generate single-season elasticity matrices and calculate λ values. The R code for this procedure is provided in Appendix S4 (Supporting Information).

At the second level of decomposition of roe deer and moose demographic processes, all survival terms of the Leslie–Usher matrices were expressed as a function of the specific predation and harvest rates affecting each age class during each of the two seasons, as defined in eqn 1. Then, elasticity values for lower-level factors were calculated using the function vitalsens in popbio (Caswell 2001; Morris & Doak 2002) and summed across seasons and age classes to obtain the overall elasticity of λ to variations in single predation or harvest rates. This allowed us to use a uniform metric to evaluate the relative impact of human harvest and of each predator species on the demography of roe deer and moose populations. Because the extent of such impact can be influenced by the predator/prey density ratio (Abrams & Ginzburg 2000), the second-level perturbation analysis was repeated with progressively increasing levels of prey density and the resulting single-predator elasticity was plotted against prey density, thus providing estimates of relative strength of predation impact on roe deer and moose populations at different predator/prey density ratios.

Finally, to more fully understand how the mechanisms of predation processes can potentially shape prey demography, predation rates were further decomposed in terms of the main factors controlling them, as defined in eqn 2, and included into the overall Leslie–Usher matrices. These factors (predator density, kill rate and the age composition of killed individuals) were functionally equivalent in the matrix, thus retaining the same total elasticity, but an evaluation of their consequences on population growth rate, under their biologically plausible range of variation, was still interesting. Therefore, a simulation approach was used to evaluate the effect of each lowest-level factor (see Fig. 1) on the demography of prey populations. For each of the two predator–prey systems, different levels of predator density, per capita kill rate and predator selectivity for adult individuals were simulated. We performed 1000 iterations for each set of predation parameters and calculated the average percentage change in λ in the prey population, using the estimated λ values derived from the baseline predator–prey system as a reference level. Confidence intervals of λ estimates were calculated as the 2·5 and 97·5 percentiles of λ distributions.

## Results

### Predation Patterns

**Fig. 2:**
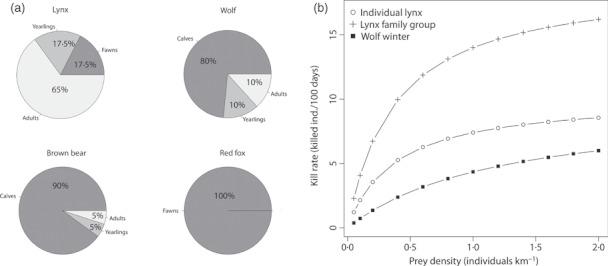
Descriptive statistics of Eurasian lynx, gray wolf, brown bear and red fox predation patterns on roe deer and moose in Scandinavia. Functional response curves (a) and age distribution of killed individuals (b) are shown. One radio-monitored adult roe deer has been killed by red fox in our study area (J.D.C. Linnell pers. comm.). However, given the insignificant demographic impact, we disregarded red fox as a predator for adult roe deer.

Based on the information derived from previously published work on Scandinavian carnivores (Sand *et al.* 2005, 2008; Andersen *et al.* 2007; Swenson *et al.* 2007; Panzacchi *et al.* 2008a, b; Nilsen *et al.* 2009b), the lynx, red fox, wolf and brown bear differ dramatically in terms of their main predation patterns. Wolf packs have a higher kill rate in summer than in winter, with an average of 54 and 26·6 moose, respectively, killed by each pack every 100 days in the two seasons, at the average moose density of our study area (Sand *et al.* 2005, 2008). When rescaling these values to a per capita kill rate, given an average wolf pack size of five individuals in Scandinavia (Sand *et al.* 2008), the resulting values are 5·3 and 10·8 killed moose per 100 days in winter and summer, respectively. Bear kill rates on moose are comparable to those of wolves, with a per capita kill rate of 6·8 individuals per 100 days (Swenson *et al.* 2007), although their predation is usually confined to a short period during spring following the birth of calves. In lynx, kill rates depend on the individual social status (Fig. 2a), with a saturation level of about 10 killed roe deer per 100 days for a solitary lynx and 19 killed by a family unit over the same time span (Nilsen *et al.* 2009b).

The four predator species also differ in terms of the age composition of killed individuals. Most (65%) of roe deer killed by lynx are adults (>2 years old), with fawns and yearlings representing the remaining 35% (Fig. 2b). Consequently, the lynx operates like a typical non-selective stalking predator, as the age composition of killed individuals is not significantly different from the overall age distribution of a roe deer population (Andersen *et al.* 2007). The wolf, brown bear, and red fox appear to be much more selective predators, as their predation comprises on average 80%, 90% and 100% fawns/calves, respectively (Sand *et al.* 2005, 2008; Panzacchi *et al.* 2008a, b), although this age class usually represents only 20–30% of roe deer and moose populations (Ericsson & Wallin 1999; Andersen *et al.* 2007).

### Perturbation Analysis (First Level) –λ and Prey Demographic Rates

The interaction among different mortality factors in roe deer and moose life cycles produced age-specific predation rates and survival estimates (Table S2, Supporting information). At the baseline densities of our study area, roe deer summer survival estimates ranged from 0·85 (95% CI = 0·82–0·87) for fawns to 0·93 (95% CI = 0·91–0·95) for adult individuals, whereas winter estimates ranged from 0·68 (95% CI = 0·65–0·73) to 0·72 (95% CI = 0·64–0·77), respectively. This resulted in overall annual survival estimates of 0·61 (95% CI = 0·57–0·69) for fawns, 0·61 (95% CI = 0·50–0·67) for yearlings, and 0·63 (95% CI = 0·60–0·68) for adults. These estimates are consistent with survival estimates of radio-collared roe deer in Norway (Nilsen *et al.* 2009a), providing an independent validation of eqns 1 and 2, and more generally of the overall demographic model. Lynx predation rates also varied between seasons and among age classes, with annual rates ranging from 0·08 (95% CI = 0·04–0·16) on fawns to 0·19 (95% CI = 0·18–0·20) on adults (Table S2, Supporting information).

As expected, moose survival estimates also varied between seasons and among age classes, with calf mortality rates being similar between seasons, and mortality among older individuals mainly occurring during the hunting season. In the baseline scenario, annual survival estimates were 0·59 (95% CI = 0·49–0·65) for calves and 0·82 (95% CI = 0·80–0·84) for adults. Moose survival estimates were also consistent with independently estimated values of annual survival in Scandinavia (Nilsen & Solberg 2006). The overall predation rate on moose calves was 0·36 (95% CI = 0·29–0·49), with wolves and bears contributing for about 66% and 34% of this value, respectively (Table S1, Supporting information), whereas predation rates on the other age classes summed to 0·04 (95% CI = 0·03–0·08), mainly due to wolf predation (75%), with the impact of brown bears on these age classes being less important (25%).

In general, moose demography was more sensitive to adult survival rates than roe deer demography (Table 1), as expected for a species with lower fecundity, older age of first reproduction and longer life expectancy (Gaillard *et al.* 2000; Nilsen *et al.* 2009a). The elasticity of roe deer λ to changes in adult survival was in fact 0·480, whereas the same parameter for the moose model was 0·736 (sum of adults and 2-year-old age classes). Conversely, the functional dependence of λ on variation in recruitment was stronger in roe deer than in moose, with corresponding elasticity values of 0·260 and 0·132, respectively.

**Table 1:** Sensitivity and elasticity values of roe deer and moose populations' λ in Scandinavia to changes in recruitment and age-specific survival rates

Symbol	Description	Sensitivity	Elasticity
Roe deer–summer			
*F*	Fecundity (includes fawn survival)	0·370	0·260
*Φ*_2,1_	Yearling survival	0·275	0·260
*Φ*_3,1_	Adult survival	0·521	0·480
Roe deer–winter			
*Φ*_1,2_	Fawn survival	0·347	0·260
*Φ*_2,2_	Yearling survival	0·379	0·260
*Φ*_3,2_	Adult survival	0·689	0·480
Moose–summer			
*F*	Fecundity (includes calf survival)	0·357	0·132
*Φ*_2,1_	Yearling survival	0·141	0·132
*Φ*_3,1_	2-year-olds survival	0·135	0·132
*Φ*_4,1_	Adult survival	0·136	0·604
Moose–winter			
*Φ*_1,2_	Calf survival	0·174	0·132
*Φ*_2,2_	Yearling survival	0·159	0·132
*Φ*_3,2_	2-year-olds survival	0·157	0·132
*Φ*_4,2_	Adult survival	0·719	0·604

Values are provided for each of the seasonal matrices, included in the periodic Leslie–Usher matrix model.

### Perturbation Analysis (Second Level) –λ and Predation Rate

**Fig. 3:**
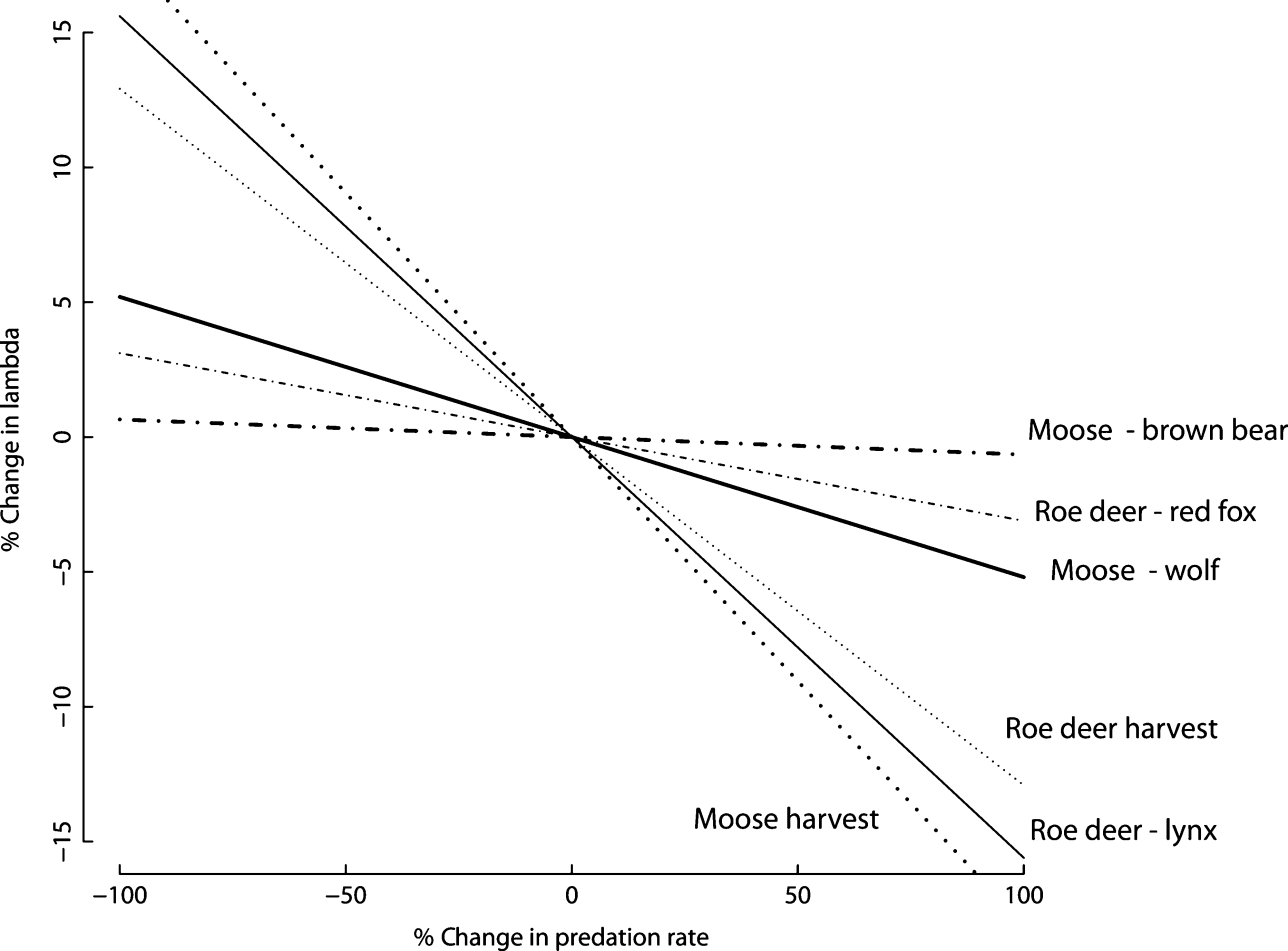
Effects of changes in predation and harvest rates of Eurasian lynx, gray wolves, brown bears, red fox and hunters on roe deer and moose population growth rate in Scandinavia, resulting from a multi-level periodic matrix model of their demography. Estimates are based on baseline densities of 0·57 and 1·00 individuals per km^2^ for roe deer and moose, respectively, and 0·4, 0·5 and 1·0 individuals per 100 km^2^ for lynx, wolf and bear, respectively. No initial density estimate was available for the red fox.

The contribution of predation rates from the four predators to changes in λ of roe deer and moose populations was remarkably different given the ecological conditions of our study area. Lynx impact on roe deer populations was by far the strongest among the four predators and was comparable to that of human harvest. Hence, a 50% increase in the lynx predation rate on roe deer corresponded to an 8% decrease in its projected growth rate, whereas a corresponding increase in the predation rates by wolves, red foxes or brown bears only generated a variation in λ between 0·3% and 2·0% (Fig. 3). Elasticity values of lynx, wolf, fox and bear predation rates were estimated at −0·157, −0·056, −0·031 and −0·006, respectively, under the baseline conditions of our study area.

**Fig. 4:**
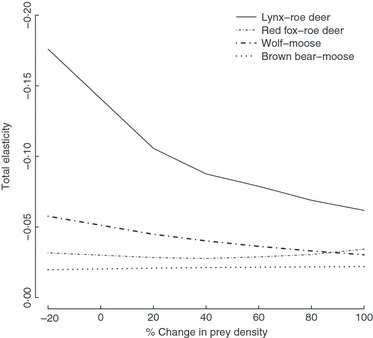
Effect of changes in prey density on the elasticity of moose and roe deer λ to Eurasian lynx, gray wolf, red fox and brown bear predation rates in Scandinavia. Baseline densities for roe deer and moose populations in the study area are 0·57 and 1·00 individuals per km^2^, respectively.

The additional perturbation analysis, conducted under progressively increasing levels of prey density, showed that the impact of lynx predation on roe deer demography decreases when prey density increases, and by doubling roe deer density, the elasticity of roe deer λ to lynx predation rate is about half of that observed in our study case (Fig. 4). Also, the elasticity of moose λ to wolf predation rate showed a decrease with increasing prey densities, but to a much lesser extent than in the roe deer–lynx system, whereas the potential impact of brown bear and red fox on prey demography did not vary significantly across different density levels (Fig. 4).

The relative impact of human harvest on roe deer and moose populations was also very different. A simulated 50% increase in harvest rate on moose caused a 10% reduction in λ, whereas the same change in roe deer harvest rate resulted in only a 5·5% reduction in roe deer population growth rate (Fig. 3). Also, while roe deer growth rate (and the resulting abundance) was significantly influenced by both human harvest and lynx predation (Fig. 3), human harvest on moose retained an overwhelming demographic impact with respect to both wolf and brown bear predation, thus substantially setting moose growth rate and density levels.

### Perturbation Analysis (Third Level) –λ and Lower-Level Predation Factors

**Fig. 5:**
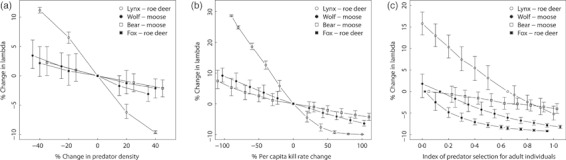
Relationship between changes in lower-level predation parameters of Eurasian lynx, gray wolf, brown bear and red fox, and the resulting projected growth rate of roe deer and moose populations in Scandinavia. The effects of changes in predator density (a), per capita kill rate (b) and predator selectivity for adult individuals (c) are shown. Points without CI bars on each line represent the baseline age selection index for each predator. Baseline densities for roe deer and moose populations in the study area are 0·57 and 1·00 individuals per km^2^, respectively, whereas for lynx, wolf and brown bear populations, they are 0·4, 0·5 and 1·0 individuals per 100 km^2^, respectively.

When simulating a change in predator density in both population models, the resulting impact of an altered scenario was different for the two prey species and for the four predators. Changes in lynx density caused large changes in the λ of roe deer population, whereas change in wolf, brown bear, and red fox densities only affected the population growth rates of their prey to a minor extent (Fig. 5a). Such a pattern was also evident when simulating a reduction in predator density in the study area. A 40% decrease in wolf, bear or fox numbers was related to about a 2–4% positive change of λ for both roe deer and moose populations, whereas the same reduction in lynx density caused a 12% increase in roe deer population growth rate (Fig. 5a).

We also observed a similar pattern when simulating a change in the per capita kill rates and in the age composition of killed individuals (Fig. 5b,c). Both roe deer and moose population growth rates were moderately influenced by per capita kill rates, as a 50% increase in the number of killed individual per 100 days by wolves and bears resulted in a 2–3% reduction in moose λ (Fig. 5b), whereas the same simulated increase in per capita kill rate in lynx was associated with a 9% reduction in roe deer λ (Fig. 5b). Nevertheless, a remarkable decrease in lynx impact on roe deer was observed when changing the age composition of their kills, while keeping the overall predation rate constant (Fig. 5c).

## Discussion

In this study, we explicitly modelled the whole process linking the predation patterns of Scandinavian large carnivores and the demography of their ungulate prey in two different predator–prey systems. By doing so, we simultaneously assessed the overall demographic impact of each predator, and the underlying nested processes determining it. To our knowledge, this is the first study to contrast differential predation strategies of carnivores within the same analytical framework, and the first using demographic elasticity as a uniform metric to compare their potential impact on prey species.

### The Role of the Four Predators in the Scandinavian Carnivore–Ungulate Trophic System

**Fig. 6:**
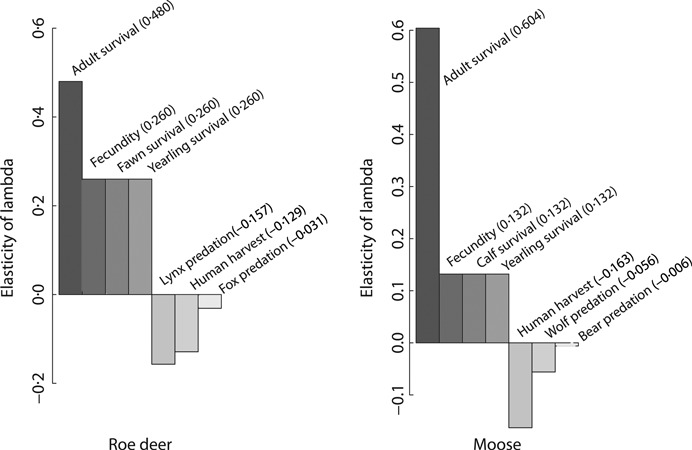
Barplot of elasticity values associated with all demographic and predation parameters, as resulting from a multi-level periodic matrix model of roe deer and moose demography in Scandinavia. Estimates are based on baseline densities of 0·57 and 1·00 individuals per km^2^ for roe deer and moose, respectively, and 0·4, 0·5, and 1·0 individuals per 100 km^2^ for lynx, wolf and bear, respectively.

The four Scandinavian predators exhibited a remarkable difference in their potential for exerting a demographic impact on prey populations, as the highest impact, lynx predation on roe deer, was about eight times higher in terms of total elasticity, than the lowest, bear predation on moose (Fig. 6).

As illustrated in Fig. 1, the overall demographic impact of predation can be viewed as the result of two nested processes: one related to the per capita impact of a given predator and resulting from its individual predation patterns and the other operating at the population level, mainly as an effect of the overall predator–prey density ratio and predation rate. In our case, the most noticeable aspect of the estimated differences in demographic impact among the four predators is the remarkable importance of per capita impact in determining its overall effect. In fact, overall predation rates did not differ greatly, ranging from 0·09 to 0·12, but produced very different demographic effects, as a consequence of different selection of prey age classes, and of the seasonal variation in their predation pressure. Similarly, predator/prey density ratios in our study area varied very little, all falling within the range of 5–7 predators/1000 individual prey, suggesting that lynx, wolf and bear exhibited a differential impact on their prey mainly as a consequence of their specific predation patterns and not simply due to numerical relations between predator and prey. This highlights the importance of per capita predation factors in the complex mechanism of predator–prey interactions, and especially the age composition of killed individuals emerged as an important underlying predictor of the overall demographic impact of predation.

It is well documented in the ecological literature that stalking predators, especially when they have a body size similar to that of their prey, are able to exert a stronger demographic control than coursing predators with a low predator–prey body size ratio (Sinclair, Mduma & Brashares 2003; Brose *et al.* 2006; Atwood, Gese & Kunkel 2007). Eurasian lynx have been shown to have impacts on roe deer densities, especially in areas of low productivity, in analyses conducted at both regional and continental scales (Melis *et al.* 2009, 2010). Therefore, the most relevant aspect of our results lies in the magnitude of the difference among the four carnivores (between three and eight times in terms of elasticity), rather than in their relative order of potential impact, and in the differential contribution of the lower-level factors (age composition of kills, kill rate, density, etc.) to the downstream demographic impact of predation.

A second result deserving attention is the relatively low demographic impact of the wolf predation on moose demography. Several studies of the wolf–moose predation system in North America have supported the hypothesis that wolves exert a substantial demographic control on their prey (Gasaway *et al.* 1992; Boertje, Valkenburg & McNay 1996; Testa 2004), even though no general agreement exists on this subject (Boutin 1992). In particular, both Gasaway *et al.* (1992) and Boertje, Valkenburg & McNay (1996) reported a substantial increase in moose density after a reduction in wolf numbers in Alaska, thus empirically providing support for the hypothesis of a wolf-controlled moose demography in their study areas. Nevertheless, these studies reported wolf/moose density ratios (0·09 and 0·009), which were about 2 and 20 times higher than that currently estimated for the Scandinavian ecosystem, respectively (Sand *et al.* 2006; Eriksen *et al.* 2008). Moreover, the proportion of moose calves in the wolf diet in Alaska was about 36% (Boertje, Valkenburg & McNay 1996), compared to 80% for the Scandinavian wolf (Sand *et al.* 2006), and average pack size was 7·5 (Ballard, Whitman & Gardner 1987), compared to about 5 in our study area (Sand *et al.* 2006). In addition to behavioural differences in cross-continental patterns of predation (Sand *et al.* 2006), these figures highlight important differences in the structure of the wolf–moose predation system between North America and Scandinavia, but also suggest that low wolf density, small pack size and the limited proportion of adult moose killed are likely the main factors explaining the low demographic impact of the recolonizing Scandinavian wolf on the moose population, at least at this early stage of its recolonization process (Wabakken *et al.* 2001). On the other hand, our results are consistent with previous explorations of the relative impacts of wolves and hunters on wild ungulate populations. In particular, Wright *et al.* (2006) found, similarly to us, that hunting exerted a greater total reproductive impact than wolves on elk populations in North America, because of the limited ability of wolves to kill a significant proportion of prime-age female elk, which have the greatest reproductive values. Such a comparison also highlights the need to identify some limitations on the generality of our findings, which are related to the specific nature of the predator–prey systems under study. Our modelling approach provides an explicit, fine-scale framework to explore the relative impact of different predators in a great variety of predator–prey systems, where the process of predation occurs in a context of wildlife management. This is a typical scenario in the majority of the European predator–prey systems and is expected to become an increasingly common situation also in the North American continent as predators expand beyond protected area networks. Nevertheless, our results cannot be directly extrapolated to unmanaged ecosystems, where the demographic interactions among predator and prey species are mediated by density dependence in vital rates, by senescence, and by the numerical response of predators to prey density, factors that were all absent in our system. Under these latter conditions, a differential relative impact of stalking vs. coursing predators can be predicted, as the above-cited factors can substantially modify the age composition of killed individuals and the ability of predators to track variations in prey density.

### The Effect of Prey and Predator Density

Our results showed that the four predators exhibited a remarkably different per capita potential impact on roe deer and moose demography. However, they also demonstrate that both predator and prey densities play an important role in modulating the overall demographic impact at the population level (Fig. 3 and 4), thus potentially modifying the relative importance of each predator and of human harvest. Lynx predation, for instance, exhibited a strong impact on roe deer at the density levels used as baseline for our study area, but its demographic impact decreased rapidly with increasing roe deer densities (Fig. 4), consistent with the findings from Melis *et al.* (2009, 2010). In contrast, red fox predation retained a constant demographic impact over the studied gradient of roe deer density, as its predation rate tended to increase at increasing roe deer densities (Panzacchi *et al.* 2008a, b). Therefore, as lynx and red fox are the main roe deer predators in several other parts of Scandinavia, where roe deer live at higher densities than in our study area, it is expected that the relative importance of the two predator species would show spatial variation along a gradient of roe deer density, with red fox playing a relatively greater role than lynx when roe deer density is higher, and vice versa at low roe deer density.

Similarly, estimates of the potential impact of wolf and bear on the moose population should not be interpreted as fixed, general traits, but rather should be evaluated in the context of the temporal and spatial variation that these impacts can exhibit. Our analyses showed that an increase in wolf density, or in the proportion of adult moose killed by wolves, can cause an increase in the overall extent of wolf impact on moose demography, and both factors should be considered in potential future scenarios, given the recolonizing nature of the Scandinavian wolf population (Wabakken *et al.* 2001). Moreover, both wolf and bear predation rates on moose are to a large extent additive with respect to human harvest and other natural mortality causes (Nilsen & Solberg 2006; Swenson *et al.* 2007). Thus, even if their per capita potential impact on moose demography is currently rather low, it operates on top of other significant mortality causes both for young and adult moose, thus requiring a coordinated planning of both wolf and bear densities objectives and moose harvest quotas in the future.

### The Mechanisms of Predation Impact and the Effect of Lower-Level Predation Patterns

Traditional studies of carnivore impact on ungulate populations have been based on experimental removal (Gasaway *et al.* 1992; Boertje, Valkenburg & McNay 1996) or on the estimation of basic predation patterns, such as kill rate (Hayes & Harestad 2000; Laundré, Hernández & Clark 2006) and predation rate (Boertje, Valkenburg & McNay 1996), as an indirect assessment of potential impact. In this study, based on the availability of two well-described predator–prey systems, we have provided a theoretical framework for the decomposition of the demographic effects of predation into their main underlying determinants, structured into three nested levels of increasing complexity. This is, to our knowledge, a novel framework, which can serve as a theoretical approach for the study of a great variety of terrestrial predator–prey systems. Even though estimating all these determinants for a specific predator–prey system might be not achievable for the majority of study cases, and indirect estimates will continue to be extensively employed in predation ecology, we stress the importance of interpreting them as single factors of a complex multi-level process. In the specific case of our study, by confirming the complex nature of predation as a driver of demographic processes in large terrestrial mammals, we suggest that kill or predation rates, if considered alone, can be poor predictors of the overall demographic impact of predation. We show that even in the same ecological context, different carnivores preying on the same prey species can exert a dramatically different demographic impact, as a direct consequence of their specific predation patterns. Further work should therefore investigate which parameters, or combinations of parameters, might be effectively used as a good predictor of the potential demographic impact of predation. The results of our study suggest that the age composition of killed individuals is likely to be strongly related to the demographic impact of predation, thus calling for a similar application of this analytical framework in other geographical and ecological contexts, to assess whether different or similar conclusions arise from other predator–prey systems. In this sense, future developments of this approach should try to include at least the two main factors that are lacking in our conceptual framework: first, to account for the effect of density-dependent factors in prey demography, which were absent in the specific conditions of our study area. Second, to assess whether the presence of a significant proportion of senescent individuals is likely to modify the potential demographic impact of different predation strategies, as different types of predators usually rely to a different extent on the old segment of a prey population. Also, substantial improvement in our understanding of predator–prey systems could be achieved in future studies by focusing on transient dynamics (sensu Mertens *et al.* 2006). Moreover, because predation patterns are the ultimate consequence of several physical and behavioural adaptations of a carnivore, such as its body size, the type and size of its social units, and its hunting strategy (Sunquist & Sunquist 1989), our results also suggest the need for a more general evaluation of the subject. It should be aimed at assessing, on a broader systematic and ecological range, how the evolutionary pressures shaping those traits can also play a role in determining the potential demographic impact of a carnivore on its prey species, and more generally the structure of ecological communities in terrestrial ecosystems.

## Supporting Information

**Appendix S1**. Estimation of roe deer absolutedensity.

**Appendix S2**. R code generating roe deer periodic matrix model.

**Appendix S3**. R code generating moose periodic matrix model.

**Appendix S4**. R code used to calculate single-season elasticity values following Caswell and Trevisan(1994).

**Fig. S1.** Average roe deer density in the study area, estimated through an independent estimate of harvest rate.

**Table S1.** Definitions and parameter values for roe deer and moose population models.

**Table S2.** Survival and predation rate estimates generated though moose and roe deer matrix models.

As a service to our authors and readers, this journal provides supporting information supplied by the authors. Such materials may be re-organized for online delivery, but are not copy-edited or typeset. Technical support issues arising from supporting information (other than missing files) should be addressed to the authors.
