# Supplementary material for: Predicting the potential demographic impact of predators on their prey: a comparative analysis of two carnivore–ungulate systems in Scandinavia
Source: J Anim Ecol. 2012 Mar;81(2):443–54. doi: 10.1111/j.1365-2656.2011.01928.x (PMC3440569; doi:10.1111/j.1365-2656.2011.01928.x)
Supplement: Supplementary file 1 [file jane0081-0443-SD1.doc]

**ONLINE SUPPORTING INFORMATION**

The following Supporting Information is available for this article online:

Appendix S1. Estimation of roe deer absolute density

Appendix S2. R code generating roe deer periodic matrix model

Appendix S3. R code generating moose periodic matrix model

Appendix S4. R code used to calculate single-season elasticity values following Caswell and Trevisan (1994)

Figure S1. Average roe deer density in the study area, estimated through an independent estimate of harvest rate.

Table S1. Definitions and parameter values for roe deer and moose population models.

Table S2. Survival and predation rate estimates generated though moose and roe deer matrix models.

Appendix S1. Estimation of roe deer absolute density.

We used an independent estimate of roe deer harvest rate based on capture-mark-recapture for our study area (J. Linnell unpublished data) and a resampling approach to transform the index of roe deer abundance (n of harvested roe deer/km^2^ per municipality into an absolute average roe deer density.

The annual roe deer harvest rate was defined through the following expression:

*HR =* $\frac{N. harvested roe deer}{N. roe deer}$ [3]

We derived harvest statistics of all municipalities in Hedmark County from Statistics Norway ([www.ssb.no](http://www.ssb.no/)), and used them to estimate the yearly average number of harvested roe deer in Hedmark and its variance during the period 1996-2005. Then, we used the R 11.2.0 software (R Development Core Team 2008) to simulate increasing levels of roe deer density, and estimated the corresponding average harvest rate through equation [3], after performing 1,000 iterations for each theoric value of roe deer density. Finally, by plotting all simulated density values against the estimated average harvest rate, we determined which one maximized the probability of observing the actual harvest rate of 0.16 estimated by Nilsen et al. (2009) of our roe deer population in Hedmark County; see Appendix Fig. S1. The ratio between the resulting density estimate and its corresponding index of roe deer abundance (n. of harvested roe deer/km^2^) was then used to obtain absolute roe deer density values for all the elements of the life cycle model.

.

Appendix S2. R code generating roe deer periodic matrix models

nf=0 # Initial number of fawns

ny=500 # Initial number of yearlings

na=2500 # Initial number of adults

nfamily=9 # N. of lynx family units observed

littersize = 2.04 # litter size

proprepro= 0.84 # proportion of females reproducing each year

fa=littersize*proprepro/2 # fecundity

prevad=0.65 # lynx index of selection for adults

prevy=(1-prevad)/2 # lynx index of slection for yearlings

prevfawn=(1-prevad)/2 # lynx index of selection for fawns

pother_s=0.01 # mortality probability for other causes in summer

pother_w=0.04 # mortality probability for other causes in winter

totalf_ratio=0.55 # adult sex ratio

prev_f=0.65 # lynx index of selection for females

area=13618 # study area

for (i in 1:nyears)

{

# SUMMER

pop0= c(nf,nf,nf,ny,ny,ny,na,na,na) # vector of population age structure
 at the beginning of summer

ntot=sum(pop0)/3 # total population size
 at the beginning of summer

d_s=((ny+na)/totalf_ratio)/area # roe deer summer density

nlynx_correct=6.24 # extrapolation index for lynx population size

nlynx=nfamily/0.22*0.78 # n. of independent lynx

a1=10.14 # lynx functional response beta values

h=0.37

kr_family_f=(prevfawn*(((a1+9.05)*(d_s))/(h+d_s)))*prev_f #lynx family kill rate on fawns

kr_family_y=(prevy*(((a1+9.05)*(d_s))/(h+d_s)))*prev_f #lynx family kill rate on yearl

kr_family_ad=(prevad*((a1+9.05)*d_s)/(h+d_s))*prev_f #lynx family kill rate on adults

kr_lynx_f=(prevfawn*((a1*(d_s))/(h+d_s)))*prev_f #lynx kill rate on fawns

kr_lynx_y=(prevy*((a1*(d_s))/(h+d_s)))*prev_f #lynx kill rate on yearlings

kr_lynx_ad=(prevad*(a1*d_s)/(h+d_s))*prev_f #lynx kill rate on adults

pfox_fawn= if (d_s <= 1.79) 0.10
 else if (d_s <= 3.57) 0.22 else 0.42 # fox predation rate on fawns

plynx_fawn_s=((kr_lynx_f*nlynx+kr_family_f*nfamily)/((na*fa)))# lynx predation rate on fawns

plynx_year_s=((kr_lynx_y*nlynx+kr_family_y*nfamily)/ny) # lynx predation rate on yearlings

plynx_ad_s=((kr_lynx_ad*nlynx+kr_family_ad*nfamily)/na) # lynx predation rate on adults

sf_s=(1-pfox_fawn)*(1-plynx_fawn_s)*(1-pother_s) # fawns summer survival

sy_s=(1-plynx_year_s)*(1-pother_s) # yearlings summer survival

sa_s=(1-plynx_ad_s)*(1-pother_s) # adults summer survival

sum_mat[1,]=c(0,0,fa*sf_s) # Leslie matrix for summer season

sum_mat[2,]=c(0,sy_s,0)

sum_mat[3,]=c(0,0,sa_s)

demo_s=pop0*sum_mat # Matrix transition process

pop1=c(sum(demo_s[1,]),sum(demo_s[1,]),sum(demo_s[1,]),

sum(demo_s[2,]),sum(demo_s[2,]),sum(demo_s[2,]),sum(demo_s[3,]),

sum(demo_s[3,]),sum(demo_s[3,]))

nf=pop1[1] # new population vectors

ny=pop1[4]

na=pop1[7]

ntot=sum(pop1)/3 # Total population size
 at the end of summer

# WINTER

d_w=(ntot/totalf_ratio)/area # winter roe deer density

kr_family_f=(prevfawn*(((a1+9.05)*(d_w))/(h+d_w)))*prev_f # lynx family kill rate on fawns

kr_family_y=(prevy*(((a1+9.05)*(d_w))/(h+d_w)))*prev_f # lynx family kill rate on yearl.

kr_family_ad=(prevad*((a1+9.05)*d_w)/(h+d_w))*prev_f # lynx family kill rate on adults

kr_lynx_f=(prevfawn*((a1*(d_w))/(h+d_w)))*prev_f #lynx kill rate on fawns

kr_lynx_y=(prevy*((a1*(d_w))/(h+d_w)))*prev_f #lynx kill rate on yearlings

kr_lynx_ad=(prevad*(a1*d_w)/(h+d_w))*prev_f #lynx kill rate on adults

plynx_fawn_w=(2.65*(kr_lynx_f*nlynx+kr_family_f*nfamily)/nf) # lynx predation rate on fawns

plynx_year_w=(2.65*(kr_lynx_y*nlynx+kr_family_y*nfamily)/ny) # lynx predation rate on yearl.

plynx_ad_w=(2.65*(kr_lynx_ad*nlynx+kr_family_ad*nfamily)/na) # lynx predation rate on adults

phunters_fawn=0.15 #harvest rate on fawns

phunters_year=0.20 #harvest rate on yearlings

phunters_ad=0.09 #harvest rate on adults

sf_w=(1-plynx_fawn_w)*(1-phunters_fawn)*(1-pother_w) # fawns winter survival

sy_w=(1-plynx_year_w)*(1-phunters_year)*(1-pother_w) # yearlings winter survival

sa_w=(1-plynx_ad_w)*(1-phunters_ad)*(1-pother_w) # adults winter survival

win_mat[1,]=c(0,0,0) #Leslie matrix for winter season

win_mat[2,]=c(sf_w,0,0)

win_mat[3,]=c(0,sy_w,sa_w)

demo_w=pop1*win_mat # Transition process

pop0=c(sum(demo_w[1,]),sum(demo_w[1,]),sum(demo_w[1,]),

sum(demo_w[2,]),sum(demo_w[2,]),sum(demo_w[2,]),
 sum(demo_w[3,]),sum(demo_w[3,]),sum(demo_w[3,]))

nf=pop0[1] # new population vectors

ny=pop0[4]

na=pop0[7]

ntot=sum(pop0)/3 # Total population size
 at the end of winter

}

Appendix S3. R code generating moose periodic matrix model

nc=0 # Initial number of fawns

ny=2500 # Initial number of yearlings

n2=1600 # Initial number of 2-years old moose

na=7500 # Initial number of adults

fa=1.2/2 # fecundity

prevcalf_s=0.90 # wolf index of selection for calves in summer

prevy_s=0.10 # wolf index of slection for yearlings in summer

prevcalf_w=0.68 # wolf index of selection for calves in winter

prevy_w=(1-prevcalf_w)/3 # wolf index of selection for yearlings in winter

prev2_w=(1-prevcalf_w)/3 # wolf index of selection for 2-years old in winter

preva_w=(1-prevcalf_w)/3 # wolf index of selection for adults in winter

pother=0.022 # mortality probability for other causes

wolf_t_size= 1000 # wolf average territoriy size

nbears=200 # bear abundance

prevcalf_b= 0.9 # bear index of selection for fawns

prevy_b= 0.05 # bear index of selection for yearlings

preva_b=0.25 # bear index of selection for 2 years old

preva_b=0.25 # bear index of selection for adults

fratio=0.5 # sex ratio

area=20000 # study area

for (i in 1:nyears)

{

# SUMMER

pop0= c(nc,nc,nc,nc,ny,ny,
 ny,ny,n2,n2,n2,n2,na,na,na,na) # vector of population age structure

ntot=sum(pop0)/4 # total population size
 at the beginning of summer

a=0.482 # wolf functional response beta values

b=1.215

d_s=(ny+n2+na+(na*fa))*2/area # moose summer density

kr_w = 54

kr_wolf_c_s=prevcalf_s*kr_w*fratio # wolf summer kill rate on fawns

kr_wolf_y_s=prevy_s*kr_w*2.30*fratio # wolf summer kill rate on yearlings

kr_bear_c=prevcalf_b*6.8*fratio # bear kill rate on calves

kr_bear_y=prevy_b*6.8*fratio # bear kill rate on fawns

kr_bear_2=prev2_b*6.8*fratio # bear kill rate on 2 years old

kr_bear_a=preva_b*6.8*fratio # bear kill rate on adults

pwolf_calf_s=(135*kr_wolf_c_s)*
 ((1/wolf_t_size)/(na*fa/area)) # wolf predation rate on calves in summer

pwolf_year_s=(135*kr_wolf_y_s)*
 ((1/wolf_t_size)/(ny/area)) # wolf predation rate on yearlings in summer

pbear_calf=(kr_bear_c*nbears)/(na*fa) # bear predation rate on calves

pbear_y=(kr_bear_y*nbears)/(ny) # bear predation rate on yearlings

pbear_2=(kr_bear_2*nbears)/(n2) # bear predation rate on yearlings

pbear_a=(kr_bear_a*nbears)/(na) # bear predation rate on adults

sc_s=(1-pbear_calf)*
 (1-pwolf_calf_s)*(1-pother) # calves summer survival

sy_s=(1-pbear_y)*
 (1-pwolf_year_s)*(1-pother) # yearlings summer survival

s2_s=(1-pbear_2)*(1-pother) # 2 years old summer survival

sa_s=(1-pbear_a)*(1-pother) # adults summer survival

sum_mat[1,]=c(0,0,0,fa*sc_s) # Leslie matrix for summer season

sum_mat[2,]=c(0,sy_s,0,0)

sum_mat[3,]=c(0,0,s2_s,0)

sum_mat[4,]=c(0,0,0,sa_s)

demo_s=pop0*sum_mat # Transition process

pop1=c(sum(demo_s[1,]),sum(demo_s[1,]),sum(demo_s[1,]),

sum(demo_s[1,]),sum(demo_s[2,]),sum(demo_s[2,]),

sum(demo_s[2,]),sum(demo_s[2,]),sum(demo_s[3,]),

sum(demo_s[3,]),sum(demo_s[3,]),sum(demo_s[3,]),

sum(demo_s[4,]),sum(demo_s[4,]),sum(demo_s[4,]),sum(demo_s[4,]))

nc=pop1[1] # new population vectors

ny=pop1[5]

n2=pop1[9]

na=pop1[13]

ntot=sum(pop1)/4 # Total population size at the end of summer

# WINTER

d_w=ntot*2/area # moose winter density

kr_wolf_c_w=prevcalf_w*(a*d_w/(b+d_w))*fratio # wolf winter kill rate on fawns

kr_wolf_y_w=prevy_w*(a*d_w/(b+d_w))*fratio # wolf winter kill rate on yearlings

kr_wolf_2_w=prev2_w*(a*d_w/(b+d_w))*fratio # wolf winter kill rate on 2 years old

kr_wolf_a_w=preva_w*(a*d_w/(b+d_w))*fratio # wolf winter kill rate on adults

pwolf_calf_w=230*kr_wolf_c_w*
 ((1/wolf_t_size)/(nc/area)) # wolf winter predation rate on calves

pwolf_year_w=230*kr_wolf_y_w*
 ((1/wolf_t_size)/(ny/area)) # wolf winter predation rate on yearlings

pwolf_2_w=230*kr_wolf_2_w*
 ((1/wolf_t_size)/(n2/area)) # wolf winter predation rate on 2 years old

pwolf_a_w=230*kr_wolf_a_w*
 ((1/wolf_t_size)/(na/area)) # wolf winter predation rate on adults

phunters_calf=0.100 # harvest rate on calves

phunters_year=0.121 # harvest rate on yearlings

phunters_2=0.106 # harvest rate on 2 years old

phunters_ad=0.130 # harvest rate on adults

sc_w=(1-pwolf_calf_w)*
 (1-phunters_calf)*(1-pother) # calves winter survival

sy_w=(1-pwolf_year_w)*
 (1-phunters_year)*(1-pother) # yearlings winter survival

s2_w=(1-pwolf_2_w)*
 (1-phunters_2)*(1-pother) # 2 years old winter survival

sa_w=(1-pwolf_a_w)*
 (1-phunters_ad)*(1-pother) # adults winter survival

win_mat[1,]=c(0,0,0,0) #Leslie matrix for winter season

win_mat[2,]=c(sc_w,0,0,0)

win_mat[3,]=c(0,sy_w,0,0)

win_mat[4,]=c(0,0,s2_w,sa_w)

demo_w=pop1*win_mat # Transition process

pop0=c(sum(demo_w[1,]),sum(demo_w[1,]),sum(demo_w[1,]),

sum(demo_w[1,]),sum(demo_w[2,]),sum(demo_w[2,]),

sum(demo_w[2,]),sum(demo_w[2,]),sum(demo_w[3,]),

sum(demo_w[3,]),sum(demo_w[3,]),sum(demo_w[3,]),

sum(demo_w[4,]),sum(demo_w[4,]),sum(demo_w[4,]),

sum(demo_w[4,]))

nc=pop0[1]

ny=pop0[5]

n2=pop0[9]

na=pop0[13]

ntot=sum(pop0)/4 # Total population size at the end of winter

}

Appendix S4. R code used to calculate single-season elasticity values following Caswell and Trevisan (1994)

**library (popbio)**

**year_mat = sum_mat%*%win_mat # create yearly matrix from the**

**product of summer**

**and winter matrices**

**trans_winmat=t(win_mat) # create a transposed**

**winter matrix**

**trans_summat=t(sum_mat) # create a transposed**

**summer matrix**

**year_sensitivity=eigen.analysis(year_mat) # create a yearly**

**sensitivity matrix**

**inverted_yearmat=win_mat%*%sum_mat # create an inverted
 yearly product matrix**

**inverted_sens=eigen.analysis(inverted_yearmat) # create sensitivity matrix**

**for the inverted matrix**

**sensitivity_summer=trans_winmat%*%inverted_sens$sensitivities # generates the summer sensitivity matrix with Caswell's method**

**sensitivity_winter=trans_summat%*%year_sensitivity$sensitivities # generates the winter sensitivity matrix with Caswell's method**

Fig. S1. Average roe deer density in Hedmark County (Norway), estimated through a resampling approach as a function of an independently derived harvest rate obtained through capture-mark-recapture analyses (J. Linnell unpublished data). Dashed lines are 95% confidence intervals of the estimate, whereas the horizontal solid line indicates the average roe deer harvest rate in Hedmark County during the period 1996-2005

**
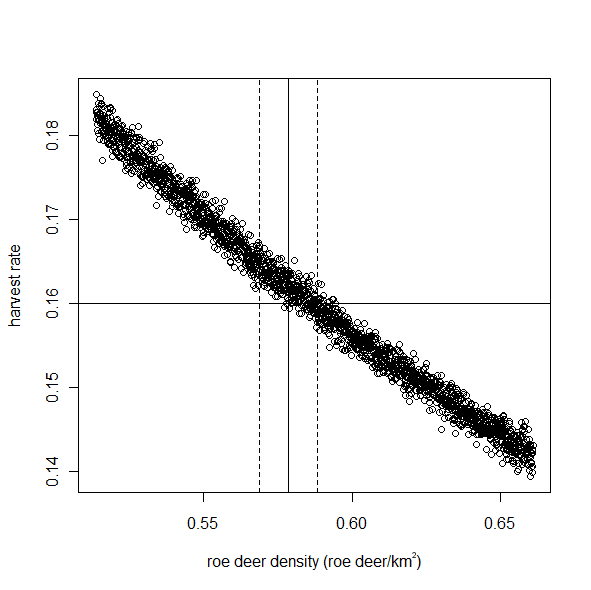
**

Table S1. Definitions and parameter values for roe deer and moose population models in Scandinavia.

| Species | Parameter | Formula | Value | Reference |
| --- | --- | --- | --- | --- |
| **Eurasian lynx** |  |  |  |  |
|  | Average n of family units observed |  | 9.0 | Linnell et al. 2010 |
|  | Proportion of adult roe deer killed |  | 0.65 | Andersen et al. 2007 |
|  | Proportion of female roe deer killed |  | 0.65 | Andersen et al. 2007 |
|  | Extrapolation factor for lynx population size |  | 6.24 | Andrén et al. 2002 |
|  | Functional response | $y=\frac{10.14x}{0.37+x}$ |  | Nilsen et al. 2009 |
| **Red fox** |  |  |  |  |
|  | Predation rates on roe deer fawns |  | 0.1, 0.22, 0.42 | J. Linnell unpublished data |
| **Roe deer** |  |  |  |  |
|  | Average litter size |  | 2.04 | Andersen & Linnell 2000 |
|  | Proportion of females reproducing each year |  | 0.84 | Andersen & Linnell 2000 |
|  | Harvest rate on fawns |  | 0.15 | J. Linnell unpublished data |
|  | Harvest rate on yearlings |  | 0.20 | J. Linnell unpublished data |
|  | Harvest rate on adults |  | 0.09 | J. Linnell unpublished data |
| **Gray wolf** |  |  |  |  |
|  | Proportion of calves killed in summer |  | 0.9 | Sand et al. 2008 |
|  | Proportion of calves killed in winter |  | 0.68 | Sand et al. 2005 |
|  | Summer kill rate |  | 54 moose/100 d | Sand et al. 2008 |
|  | Winter functional response | $y=\frac{0.482x}{1.215+x}$ |  | Sand et al. (unpublished data) |
|  |  |  |  |  |
| **Brown bear** |  |  |  |  |
|  | Proportion of moose calves killed |  | 0.9 | Swenson et al. 2007 |
|  | Kill rate (killed moose/100 days) |  | 6.8 | Swenson et al. 2007 |
| **Moose** |  |  |  |  |
|  | Fecundity |  | 1.2 | Ericsson & Wallin 2010 |
|  | Harvest rate on calves |  | 0.1 | Ericsson & Wallin 2001 |
|  | Harvest rate on yearlings |  | 0.121 | Ericsson & Wallin 2001 |
|  | Harvest rate on 2 years old |  | 0.106 | Ericsson & Wallin 2001 |
|  | Harvest rate on adults |  | 0.130 | Ericsson & Wallin 2001 |

Table S2. Survival and predation rate estimates generated through Scandinavian moose and roe deer matrix models . Figures within brackets are 95% CIs. Estimates are based on baseline densities of 0.57 and 1.00 individuals/km^2^ for roe deer and moose, respectively, 0.4, 0.5, and 1.0 individuals/100 km^2^ for lynx, wolf, and bear, respectively. No initial density estimate was available for the red fox.

| Parameter | Description | Summer | Winter | Annual |
| --- | --- | --- | --- | --- |
| **Roe deer** |  |  |  |  |
| *Φ_1_* | Fawn survival | 0.85 (0.82-0.87) | 0.72 (0.64-0.77) | 0.61 (0.57-0.69) |
| *Φ_2_* | Yearling survival | 0.94 (0.90-0.96) | 0.64 (0.56-0.70) | 0.61 (0.50-0.67) |
| *Φ_3_* | Adult survival | 0.93 (0.91-0.95) | 0.68 (0.65-0.73) | 0.63 (0.60-0.68) |
| *PR(lynx) _1_* | Lynx predation rate on fawns | 0.03 (0.01-0.05) | 0.11 (0.05-0.20) | 0.08 (0.04-0.16) |
| *PR(lynx) _2_* | Lynx predation rate on yearlings | 0.05 (0.03-0.08) | 0.15 (0.08-0.25) | 0.10 (0.07-0.13) |
| *PR(lynx) _3_* | Lynx predation rate on adults | 0.07 (0.05-0.08) | 0.21 (0.16-0.24) | 0.19 (0.18-0.20) |
| *PR(fox)_1_* | Red fox predation rate on fawns | 0.12 (0.11-0.19) | - | 0.12 (0.11-0.19) |
| **Moose** |  |  |  |  |
| *Φ_1_* | Calf survival | 0.77 (0.68-0.82) | 0.77 (0.71-0.80) | 0.59 (0.49-0.65) |
| *Φ_2_* | Yearling survival | 0.95 (0.93-0.96) | 0.83 (0.81-0.84) | 0.79 (0.74-0.80) |
| *Φ_3_* | 2-years old survival | 0.97 (0.95-0.98) | 0.81 (0.80-0.83) | 0.79 (0.77-0.80) |
| *Φ_4_* | Adult survival | 0.98 (0.96-0.98) | 0.84 (0.83-0.85) | 0.82 (0.79-0.84) |
| *PR(wolf) _1_* | Wolf predation rate on calves | 0.14 (0.11-0.20) | 0.12 (0.08-0.18) | 0.24 (0.18-0.33) |
| *PR(wolf) _2_* | Wolf predation rate on yearlings | 0.03 (0.02-0.04) | 0.03 (0.02-0.04) | 0.05 (0.05-0.06) |
| *PR(wolf) _3,4_* | Wolf predation rate on adults | - | 0.03 (0.02-0.05) | 0.03 (0.02-0.05) |
| *PR(bear) _1_* | Bear predation rate on calves | 0.12 (0.11-0.16) | - | 0.12 (0.11-0.16) |
| *PR(bear) _2,3.4_* | Bear predation rate on other age classes | 0.01 (0.01-0.03) | - | 0.01 (0.01-0.03) |

**SUPPORTING INFORMATION** **REFERENCES**

Andersen, R., Karlsen, J., Austmo, L.B., Odden, J., Linnell, J.D.C. & Gaillard, J.M. (2007) Selectivity of Eurasian lynx Lynx lynx and recreational hunters for age, sex and body condition in roe deer Capreolus capreolus. *Wildlife Biology*, **13**, 467-474.

Andersen, R. & Linnell, J.D.C. (2000) Irruptive demography in roe deer: density dependent effects on body mass and fertility. *Journal of Wildlife Management*, **64**, 698-706.

Andrén, H., Linnell, J.D.C., Liberg, O., Ahlqvist, P., Andersen, R., Danell, A., Kvam, T., Odden, J. & Segerström, P. (2002) Estimating total lynx Lynx lynx population size from censuses of family groups. *Wildlife Biology*, **4**, 299-306.

Caswell, H. & Trevisan, M.C. (1994) Sensitivity analysis of periodic matrix models. *Ecology*, **75**, 1299-1303.

Ericsson, G. & Wallin, K. (2001) Age-specific moose (Alces alces) mortality in a predator-free environment : Evidence for senescence in females. *Ecoscience*, **8**, 157-163.

Ericsson, G., Wallin, K., Ball, J.P. & Broberg, M. (2001) Age-related reproductive Effort and senescence in free-ranging moose, Alces alces. *Ecology*, **82**, 1613-1620.

Linnell, J.D.C., Broseth, H., Odden, J. & Nilsen, E.B. (2010) Sustainably harvesting a large carnivore? Development of Eurasian lynx populations in Norway during 160 years of shifting policy. *Environmental Management*, **45**, 1142-1154.

Nilsen, E.B., Linnell, J.D.C., Odden, J. & Andersen, R. (2009) Climate, season, and social status modulate the functional response of an efficient stalking predator: the Eurasian lynx. *Journal of Animal Ecology*, **78**, 741-751.

R Development Core Team (2008) *R: A language and environment for statistical computing*. R Foundation for Statistical Computing, Vienna, Austria.

Sand, H., Wabakken, P., Zimmermann, B., Johansson, O., Pedersen, H.C. & Liberg, O. (2008) Summer kill rates and predation pattern in a wolf – moose system: can we rely on winter estimates? *Oecologia*, **156**, 53-64.

Sand, H., Zimmermann, B., Wabakken, P., Andrèn, H. & Pedersen, H.C. (2005) Using GPS technology and GIS cluster analyses to estimate kill rates in wolf—ungulate ecosystems. *Wildlife Society Bulletin*, **33**, 914-925.

Sunquist, M. E., and F. C. Sunquist (1989) Ecological constraints on predation by large felids. Pages 382–409 in J. L. Gittleman, editor. Carnivore behavior, ecology, and evolution. Cornell University Press, Ithaca, New York, USA.

Swenson, J.E., Dahle, B., Busk, H., Opseth, O., Johansen, T., Söderberg, A., Wallin, K. & Cederlund, G. (2007) Predation on moose calves by European brown bears. *Journal of Wildlife Management*, **71**, 1993-1997.
